# NF-κB activation in myeloid cells mediates ventilator-induced lung injury

**DOI:** 10.1186/1465-9921-14-69

**Published:** 2013-07-03

**Authors:** Yi-An Ko, Ming-Chieh Yang, Hung-Tu Huang, Ching-Mei Hsu, Lee-Wei Chen

**Affiliations:** 1 Department of Biological Sciences, National Sun Yat-Sen University, 70 Lien-Hai Road, Kaohsiung 804, Taiwan; 2 Department of Surgery, Kaohsiung Veterans General Hospital, Ta-chung 1st Road, Kaohsiung 386, Taiwan; 3 Department of Anatomy, School of Medicine, Kaohsiung Medical University, Kaohsiung, Taiwan; 4 Institute of Emergency and Critical Care Medicine, National Yang-Ming University, Taipei, Taiwan

**Keywords:** Mechanical ventilator, Inflammation, IL-6, NF-κB, Alveolar macrophage, Chimeric mice

## Abstract

**Background:**

Although use of the mechanical ventilator is a life-saving intervention, excessive tidal volumes will activate NF-κB in the lung with subsequent induction of lung edema formation, neutrophil infiltration and proinflammatory cytokine/chemokine release. The roles of NF-κB and IL-6 in ventilator-induced lung injury (VILI) remain widely debated.

**Methods:**

To study the molecular mechanisms of the pathogenesis of VILI, mice with a deletion of IкB kinase in the myeloid cells (IKKβ^△mye^), IL-6^-/-^ to WT chimeric mice, and C57BL/6 mice (WT) were placed on a ventilator for 6 hr.

WT mice were also given an IL-6-blocking antibody to examine the role of IL-6 in VILI.

**Results:**

Our results revealed that high tidal volume ventilation induced pulmonary capillary permeability, neutrophil sequestration, macrophage drifting as well as increased protein in bronchoalveolar lavage fluid (BALF). IL-6 production and IL-1β, CXCR2, and MIP2 expression were also increased in WT lungs but not in those pretreated with IL-6-blocking antibodies. Further, ventilator-induced protein concentrations and total cells in BALF, as well as lung permeability, were all significantly decreased in IKKβ^△mye^ mice as well as in IL6^-/-^ to WT chimeric mice.

**Conclusion:**

Given that IKKβ^△mye^ mice demonstrated a significant decrease in ventilator-induced IL-6 production, we conclude that NF-κB–IL-6 signaling pathways induce inflammation, contributing to VILI, and IкB kinase in the myeloid cells mediates ventilator-induced IL-6 production, inflammation, and lung injury.

## Background

Mechanical ventilators (MV) are used to assist ill patients with respiratory failure to retain normal ventilatory pumping, pulmonary gas exchange, and avoid alveolar collapse [1]. Longer ventilation time and excessive tidal volume have been shown to contribute to lung injury [2-4] and are associated with higher mortality [5]. Human studies suggest that the release of cytokines/chemokines and the recruitment of leukocytes causes ventilator-associated lung injury (VALI) [6]. Experimental models have demonstrated increased vascular permeability, higher cell count and protein concentration in the bronchoalveolar lavage fluid (BALF), and increased inflammatory cell infiltration into lung tissues in ventilator-induced lung injury (VILI) [1,2,6-8]. Thus, ventilator stress damages the alveolar barrier and facilitates leukocyte infiltration to promote an inflammatory response.

NF-κB, a heterodimer composed of p50/p65, acts as a nucleoprotein that binds to DNA and regulates the genes encoding proinflammatory cytokines/chemokines, adhesion molecules, as well as the regulatory factors in cell cycle and survival [9,10]. Proteolytic degradation of IκB that has been phosphorylation by IκB kinase (IKK) liberates NF-κB to enter the nucleus and activates the NF-κB-regulated target genes. This process is eventually terminated through the NF-κB-induced synthesis of IκBs and, consecutively, cytoplasmic resequestration of this transcription factor. Previous study has demonstrated that both hyperoxia and overventilation would activate NF-κB with subsequent induction of lung edema formation, neutrophil infiltration and proinflammatory cytokines/chemokines release. Studies also showed that the potent inhibitor of NF-κB and steroid could reduce the injury of ventilation [11,12]. The effects of NF-κB activation in the cellular level under the stimulation of ventilation remain poorly understood.

Interleukin-6 (IL-6) is a pleiotropic cytokine involved in both pro-inflammatory and anti-inflammatory responses via regulating leukocyte function and apoptosis [13]. IL-6 is a protective factor that decreases the injury produced by the shock model, pulmonary inflammation, and oxidative damage [8,14,15]. Furthermore, alveolar barrier disruption and lung permeability can be improved by neutrophil-derived IL-6 in VILI [8]. However, patients with lower plasma levels of IL-6 were associated with better outcome [16] and had a lower risk of developing ventilator-associated pneumonia [17]. Therefore, the precise role of IL-6 in VILI is still debatable.

Other cytokines produced by bronchial, bronchiolar and alveolar epithelial cells as well as alveolar macrophages and neutrophils, have also been shown to be important for signaling between inflammatory cells and recruiting leucocytes to the lung [6]. The cytokines IL-1 and TNF-α activate NF-κB, resulting in transcription of genes necessary for the innate immune response [6,9]. CXC chemokines, inter-cellular adhesion molecule (ICAM) and vascular cell adhesion protein (VCAM), regulated by IL-6 and IL-1, can facilitate neutrophil infiltration [6,13,18], and MIP-2 is a potent leukocyte chemoattractant [6]. The levels of proinflammatory cytokines in lung homogenates, serum and BALF have also been found to be increased after ventilation in both clinical and animal models [5,6,19]. The increased production of cytokines/chemokines is critical for the pathogenesis of VILI and the increased pulmonary vascular leakage that allows leukocytes to enter the tissue spaces and induces inflammation.

The mechanisms and interactions of IL-6 and NF-κB in the pathogenesis of VILI are still elusive. We hypothesized that high stretch ventilation stimulated NF-κB activation of alveolar macrophages that induced IL-6 and subsequent IL-1β, CXCR2, as well as MIP2 expression in the lung and finally the inadvertent activation of inflammation. Using a high tidal volume ventilation model in mice, we demonstrate that ventilator-induced IL-6 production and lung permeability were decreased in IKKβ^△mye^ mice when compared with WT mice. This suggests that nuclear factor-κB activation in myeloid cell mediates ventilator-induced IL-6 production as well as lung injury. Using a NF-κB inhibitor to decrease IL-6 production in the lung and subsequent VILI might be a useful strategy in critical patients.

## Methods

### Animals

Specific pathogen–free C57BL/6 mice weighing between 20 and 25 g were purchased from the National Laboratory Breeding and Research Center (NLBRC, Taipei, Taiwan). Mice genetically deficient for IL-6, (IL-6^-/-^, in the C57/BL/6 genetic background) were purchased from The Jackson Laboratory (Bar Harbor, ME). We obtained LysM-Cre mice from the Jackson laboratory that express Cre recombinase from the endogenous *Lyzs* locus. We crossed these mice with a strain containing a *loxP* site flanking IKKβ previously obtained from Dr. Karin’s lab at University of California in San Diego. Cre-mediated recombination results in deletion of the IKKβ gene in the myeloid cell lineage, including monocytes, mature macrophages, and granulocytes as previously described [20]. All purchased animals were maintained in a temperature and diet controlled room for at least 1 week before the experiments. All animal procedures were in compliance with regulations on animals used for experimental and other scientific purposes approved by the National Sun Yat-Sen University Animal Experiments Committee.

### Experimental design

Since ventilation-induced stretch results in lung damage, we established an animal model to investigate the possible mechanisms of VILI. WT mice ventilated with low or high tidal volumes for 6 hr without positive end expiratory pressure (PEEP) were assayed for pulmonary vascular permeability and neutrophil accumulation. Pulmonary vascular leakage was quantified by measuring the extravasation of EBD and lung MPO activity, representing neutrophil infiltration into the vasculature and alveoli of the lung. The BALF was also collected to evaluate the extent of lung damage.

In experiment 1, C57BL/6 (wild-type, WT) mice were divided into three groups. Group I (control group, n = 6), received anesthesia, tracheostomy, and endotracheal intubation for six hours. Group II (low tidal volume group, n = 6), received anesthesia, tracheostomy, and endotracheal intubation with low tidal volume ventilation for six hours. Group III (high tidal volume group, n = 6), received anesthesia, tracheostomy, and endotracheal intubation with high tidal volume ventilation for six hours. Lung tissues were harvested to assay injury, expression of proinflammatory cytokines/chemokines, NF-κB DNA-binding activity, and morphology. Bronchoalveolar lavage fluid (BALF) was also collected for cell counting and cytokine assay.

In experiment 2, mice with deletion of IкB kinase in myeloid cells (IKKβ^△mye^) were divided into two groups. Group I (control group, n = 6) received anesthesia, tracheostomy, and endotracheal intubation for six hours. Group II (high tidal volume group, n = 6) received anesthesia, tracheostomy, and endotracheal intubation with high tidal volume ventilation for six hours. The lung tissues were harvested and assayed as above.

In experiment 3, a specific antibody for IL-6 (R&D systems, Minneapolis, MN) was given (0.25 mg/kg, i.p.) to WT mice just before ventilator treatment and the effects of IL-6 blocking was evaluated by the assays described in experiment 1. C57BL/6 (wild-type, WT) mice were divided into three groups. Group I (control group, n = 6), received vehicle treatment, anesthesia, tracheostomy, and endotracheal intubation for six hours. Group II (high tidal volume group, n = 6), received vehicle treatment, anesthesia, tracheostomy, and endotracheal intubation with high tidal volume ventilation for six hours. Group III (high tidal volume + IL-6 Ab group, n = 6), received IL-6 antibiotic treatment, anesthesia, tracheostomy, and endotracheal intubation with high tidal volume ventilation for six hours. The lung tissues were harvested and assayed as above.

In experiment 4, to study whether the myeloid or resident cells play a critical role in VILI, the bone marrow cells of WT and IL6^-/-^ mice were harvested and injected into WT mice respectively to generate the chimeric mice (WT to WT and IL6^-/-^ to WT mice). Bone marrow-transplanted chimeras are represented in the format of bone marrow donor to bone marrow recipient (e.g., IL6^-/-^ to WT). Six to eight weeks after transplantation, animals (n = 6 for each group) were subjected to high tidal volume ventilation treatment and the lung tissues and BALF were harvested for examination.

### Ventilator-induced lung injury in a mouse model

Mice were anaesthetized intraperitoneally with ketamine (80 mg/kg) and xylazine (10 mg/kg), and the nuchal skin was cut 1 cm below the mouth. Muscles were separated and the trachea was exposed and cannulated with 0.7 cm 21G flat syringe needle that connected to a mechanical ventilator (SAR-830/P, CWE, Pennsylvania, USA) for 6 hr. During the period of mechanical ventilation, the mice were given Avertin (15 mg/kg) and supplied with sterile saline (10 μl/g) every hour. The ventilation strategy was low stretch (tidal volume = 10 ml/kg) or high stretch (tidal volume = 30 ml/kg) and without end expiratory pressure (PEEP). The control mice breathed spontaneously during this 6-hour period.

### Quantification of pulmonary microvascular injury

Ventilation-induced pulmonary microvascular dysfunction was quantified by measuring the concentration of Evans Blue Dye (EBD) within the lung after intravenous injection of the dye. EBD binds avidly to albumin and has been used as a marker of protein extravasation in models of inflammatory tissue injury [21]. EBD was injected (30 mg/kg) into the femoral vein 10 min before the termination of experiment. The thoracic cavity was opened and blood samples (0.1 ml each) were taken from heart after infusion and centrifuged at 2,400 × g for 7 min to collect the plasma. The pulmonary vasculature was cleared of blood by flushing 3 ml saline through left ventricular and the lungs were weighed, placed in 1 ml of formamide and incubated at 37°C for 16 hr. The concentrations of EBD extracted from both lung and plasma were measured at 620 nm. The absorbance at 740 nm was subtracted from the 620 nm absorbance values to deduct the contributions from hemoglobin contamination. The concentration of EBD in lung was normalized by using the formula: (lung A_620_/g lung/plasmaA_620_) [7] and presented as permeability index.

### Pulmonary neutrophil infiltration assay

Lung myeloperoxidase (MPO) activity has been used as a maker of lung neutrophil infiltration [11]. Mice were anesthetized and the thorax was opened with median sternotomy. The bilateral lung and heart were harvested together, and the pulmonary vasculature was cleared of blood by gently injecting of 5 ml saline into the right ventricle. The lung were blotted dry of surface blood and weighed.

Lung tissues were placed in 50 mM potassium phosphate buffer (pH 6.0) with 0.5% hexadecyltrimethylammonium bromide and homogenized. The homogenate was centrifuged at 9500 × g, 4°C for 10 min. An aliquot (60 μl) of supernatant was added to 939 μl of potassium phosphate buffer (pH 6.0) with 16.7 mg/ml of *O*-dianisidine and 0.5% hydrogen peroxide. The rate of change in absorbance at 460 nm was measured over 2 min. One unit of MPO activity was defined as the amount of enzyme that reduced 1 μmole of peroxide per min and the data were expressed as units per gram of lung tissue (Units/g tissue).

### Preparation of bronchoalveolar lavage fluid (BALF)

MV is thought to contribute to the monocyte/macrophage drift in the tracheobronchial space [7], which can be measured by analysis of BALF. For whole lung lavage, the lavage was washed 6 times with 2 separate injections of 0.5 ml sterile saline through a 21G flat syringe needle which was cannulated 0.7 cm into the trachea. BALF collected was used for quantitative cell counting with a hemocytometer. The BALF was also centrifuged at 350 × g for 5 min, and the supernatant stored at -80°C for cytokine analysis [6].

### Western immunoblots

The harvested lung tissue was weighed and homogenized in protein extraction buffer (Sigma) containing proteinase inhibitor cocktail (Roche), 1 mM NaF and 1 mM Na_3_VO_4_. Homogenized samples (100 μg of protein each) were subjected to SDS-PAGE at 50 to 80 V for 3 hr. Proteins were transferred onto a nitrocellulose membrane and the membrane was blocked with 5% non-fat milk in TBST buffer (10 mM Tris–HCl, pH 7.5, 150 mM NaCl and 1.2% Tween 20) at room temperature for 1 hr and incubated with antibody against IL-6, IL-1β, and ICAM at room temperature for 1 hr. After immunoblotting with the specific primary antibodies, membranes were washed with TBST and incubated with the secondary antibody at room temperature for 1 hr. The membranes were washed with TBST and the protein bands were detected by enhanced chemiluminescence (ECL) detection reagent (Millipore).

### Reverse transcription polymerase chain reaction (RT-PCR)

The total RNAs were extracted from lung tissue and cells in BALF using the Miniprep Purification Kit (GeneMark). The cDNAs encoding proinflammatory cytokines/chemokines were generated by reverse transcription and amplified by PCR. Sets of IL-6, IL-1β, ICAM, CXCR2, MIP-2, and GADPH (Glyceraldehyde-3-phosphate dehydrogenase) primers were designed according to those genes documented in GenBank.

For the PCR reaction, to the sterile 0.2 ml tube were added 3 μl of 10× Gene Taq buffer (Gene Mark Inc. Atlanta, USA), 2 μl of 2.5 mM dNTP, 0.5 μl of 25 mM sense and anti-sense primers, and an appropriate amount of water to make a total volume of 30 μl. After adding 0.05 μl of Gene Taq DNA polymerase (5U/μl), amplification was performed in a thermocycler (Bio-Rad) with the following profile: 5 min at 95°C before the first cycle, 1 min at 95°C for denaturation, 1 min at 58°C for annealing, and 1 min 30 sec at 72°C for extension, finally 10 min at 72°C after the last cycle. The PCR products were separated on 1.5% agarose gel and stained with ethidium bromide. The approximate size of the PCR product was obtained by comparing with the markers (100 bp Ladder, Biolads).

### Enzyme-linked immunosorbent assay (ELISA)

The lung tissue, BALF, and supernatant of LPS-stimulated macrophages were collected for IL-6, IL-1β, and TNF-α assay by using the mouse ELISA kit (eBioscience). Tissues were homogenized in lysis buffer containing 30 mM Tris, pH 7.5, 300 mM NaCl, 2 mM MgCl_2_, 10% Triton X-100, 2 mM CaCl_2_, and 20 μg/ml of pepstatin A/leupeptin/aprotinin. The homogenate was centrifuged at 1,000 × g, 4°C for 15 min and the supernatant was collected for use. The ELISA plates were coated with 100 μl capture antibody per well at 4°C overnight. After appropriate wash, 200 μl of assay dilution buffer was added per well for blocking at room temperature for 1 hr. The sample and serial dilutions of standards were added to the plate and incubated at 4°C overnight. After coating with detection antibody, avidin-HRP was added and incubated at room temperature for 30 min. The substrate 3,3′,5,5′-tetramethylbenzidine (TMB) was added and incubated for 15 min. Finally, 2 N H_2_SO_4_ was added to stop the reaction and absorbance at 450 nm was measured using an ELISA reader.

### Electrophoretic mobility shift assay for NF-κB

Tissue nuclear extract was obtained by using NE-PER nuclear and cytoplasmic extraction reagents (CER I, CER II and NER, Pierce). Tissue was added 100 μl of ice-cold CER I containing proteinase inhibitors (0.5 mM PMSF, 1 mM pepstatinA, 1 mM leupeptin, 1 mM aprotinin, 1 mM NaF, and 1 mM Na_3_VO_4_). Sample was vortexed vigorously for 15 sec to fully resuspend the tissue and incubated on ice for 10 min. The mixture was added 11 μl of ice-cold CER II, vortexed for 5 sec, and incubated on ice for 1 min, then vortexed for 5 sec, and finally centrifuged at 4°C, 9,500 × g for 7 min. The supernatant fraction (cytoplasmic extract) was stored and the insoluble fraction containing nuclei was resuspended in 50 μl of NER. The suspension was incubated on ice and vortexed for 15 sec every 10 min, for a total of 40 min. Finally the suspension was centrifuged at 4°C, 9,500 × g for 12 min and the supernatant fraction (nuclear extract) was immediately transferred to a clean tube and stored at -80°C until use.

The Bandshift kit (Promega) was used according to the manufacturer’s instructions. The double-stranded oligonucleotide DNA probe containing the NF-κB binding consensus sequence (5′AGTTGAGGGGAC-TTTCCCAGGC3′) was end-labeled with [γ-^32^P]ATP (3,000 Ci/mmol at 10 mCi/ml) at 37°C for 10 min using polynucleotide kinase (5U/μl). To remove the free radionucleotides, centrifugation with the G-25 microspin column (Promega) at 600 × g for 5 min was carried out. The probes were incubated with 5 μg of tissue nuclear proteins in gel shift binding buffer containing 10 mM Tris–HCl (pH 7.5), 50 mM NaCl, 1 mM MgCl_2_, 0.5 mM DTT, 0.5 mM EDTA, 20% glycerol and 0.5 mg/ml of poly (dI-dC) to reduce nonspecific binding. Incubations were carried out at room temperature for 30 min. Reaction mixtures were electrophoresed at 4 °C, 160 V on a 4% nondenaturing polyacrylamide gel and autoradiographed at -80 °C for 12 to 48 hr.

### Ex vivo alveolar macrophage stimulation

Alveolar macrophages (AM) were harvested from adult mice by bronchoalveolar lavage (BAL) with Tris-buffered saline containing 0.25 mM EDTA and EGTA. Cells were resuspended in RPMI 1640 in a final concentration of 1 × 10^5^ cells/ml. Cells were then cultured in 96-well microtiter plates for 2 h and washed with RPMI 1640 to remove nonadherent cells [22]. Adherent monolayer cells were stimulated with different doses of LPS (from *Escherichia coli* O26:B6 Sigma-Aldrich) or RPMI 1640 for 4 h. Supernatants were collected and stored at -70°C until assayed for TNF-α.

### Histology

The lung samples were collected and fixed in 4% formalin. The samples were embedded in paraffin, cut into 3–5 μm sections, and stained with haematoxylin and eosin. Pulmonary edema and the infiltration of inflammatory cells were observed.

### Chimeric mice

Adoptive transfer of myeloid cells using bone marrow cells has become a well-accepted method for establishing the contribution of hematopoietic or non-hematopoietic cells [23]. Bone marrow cells were harvested from femur and tibia bones of WT or IL-6^-/-^ mice. Recipient animals were lethally irradiated with 950 cGy using a ^137^Cs source twice with a one-hour interval. Bone marrow cells (at least 1 × 10^6^ cells) were suspended in 100 μl saline and injected into the tail vein of an 8-week-old recipient. The chimeric mice were used for experiments after 4–6 weeks. To confirm the success of adoptive transfer, the genotype of bone marrow cells in chimera was determined after experiments and showed successful reconstitution (data not shown).

### Statistics

All data are analyzed by one-way analysis of variance (ANOVA), followed by Tukey’s Multiple Comparison Test. All values in the figures and text were expressed as mean ± standard error of the mean, and P values of less than 0.05 are considered to be statistically significant.

## Results

### Ventilator induced lung injury in WT mice

**Figure 1:**
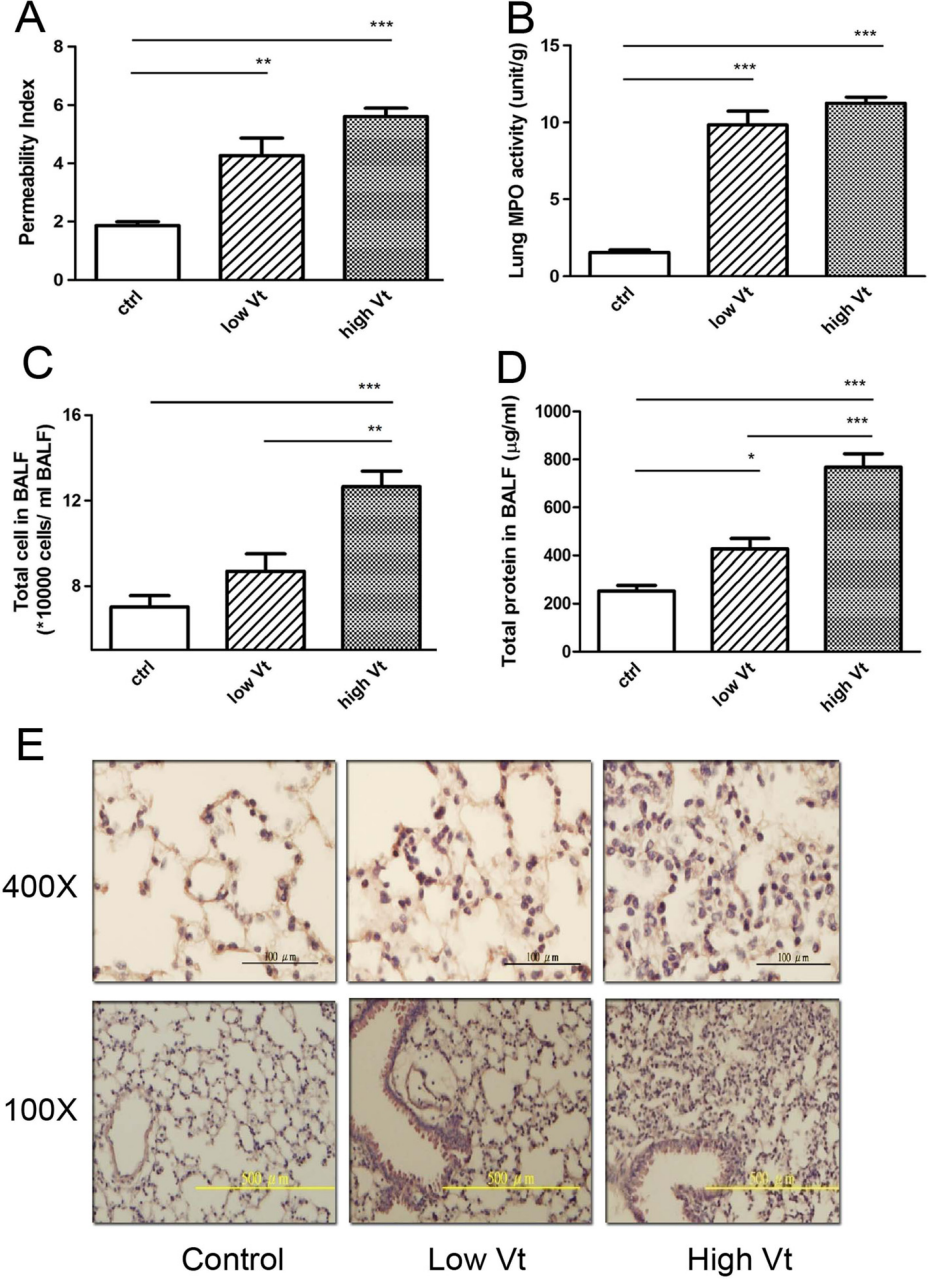
**Mechanical ventilation induced lung injury.** The microvascular permeability was increased in ventilator treated WT mice as assayed with extravasation Evans Blue dye **(A)**. The increased neutrophil infiltration **(B)** was demonstrated by increased myeloperoxidase (MPO) activity in the lung of WT mice with ventilator treatment. Total cell counts **(C)** and total protein concentration **(D)** in BALF were increased in WT mice with ventilator treatment. *P<0.05, **P<0.01, ***P<0.001. **(E)**. The extent of neutrophil infiltration and alveolus swelling was observed in WT mice with low tidal volume (Low Vt) or high tidal volume (High Vt) ventilator treatment with HE stain.

WT mice demonstrated a significant increase in pulmonary vascular permeability (Figure 1A) and MPO activity (Figure 1B) after ventilation when compared with the control group and the extent of the increase was higher in the high tidal volume group. Also, ventilation with low and high tidal volume caused 1.2- and 1.8-fold increases in the total number of cells (Figure 1C) as well as 1.7- and 3-fold increases in total protein concentration (Figure 1D) in BALF of mice with low and high tidal volume ventilator treatment, respectively, when compared with the control group.

The effects of ventilation on lung morphology were also examined by histological evaluation of tissue sections. Our data demonstrated that ventilator-induced inflammatory cell infiltration, swelling of the parenchyma as well as alveoli and the extent of cell infiltration were enhanced with increased tidal volume (Figure 1E).

### Ventilator induced NF-κB activation and production of IL-6, IL-1β, and ICAM in the lung

**Figure 2:**
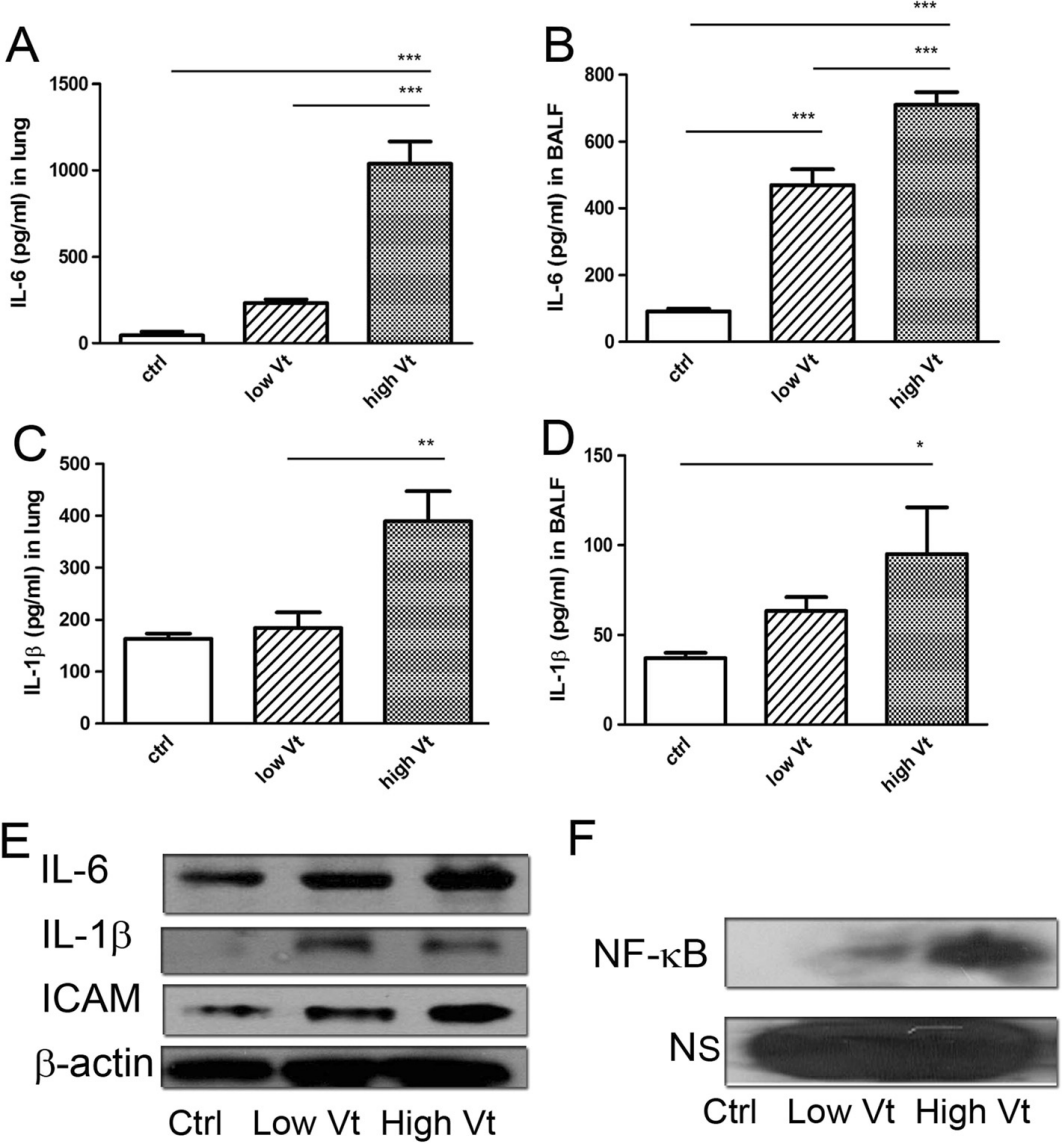
**The mechanical ventilator (MV) induced the levels of IL-6 and IL-1β in the lung and BALF.** MV significantly increased the production of IL-6 **(A, B)** and IL-1β **(C, D)** in the lung and BALF, respectively. *P<0.05, **P<0.01, ***P<0.001. **(E)** Low tidal volume (Low Vt) or high tidal volume (High Vt) ventilator treatment induced IL-6, IL-1β, and ICAM protein expression in the lung in WT mice. **(F)** NF-κB DNA binding activity of the lung was increased with the strength of ventilation in WT mice. NS=non-specific binding.

To study the effect of ventilators on NF-κB activation and IL-6 and IL-1β levels in the lung, lung homogenates were examined. High tidal volume ventilation induced 23- and 8-fold increases in IL-6 levels in the lung and BALF, respectively, as compared to the control group (Figure 2A, 2B). Ventilator-induced IL-6 levels were much greater than IL-1β levels (Figure 2C, 2D). Notably, levels of IL-6 were already significantly increased in BALF in WT mice with low tidal volume ventilation treatment. The protein levels of IL-6, IL-1β, and ICAM (Figure 2E) were also increased in WT mice with ventilator treatment.

NF-κB is a broad gene transcription regulatory protein and its activation has been observed in the animal VILI model. Similar to the cytokine production, DNA binding activity of NF-κB in the lung was increased in ventilator treated mice as demonstrated by EMSA. The extent of NF-κB activation was greater in the high tidal volume group than the low tidal volume group (Figure 2F).

### Ventilators did not induce lung injury in IKKβ^△mye^ mice

**Figure 3:**
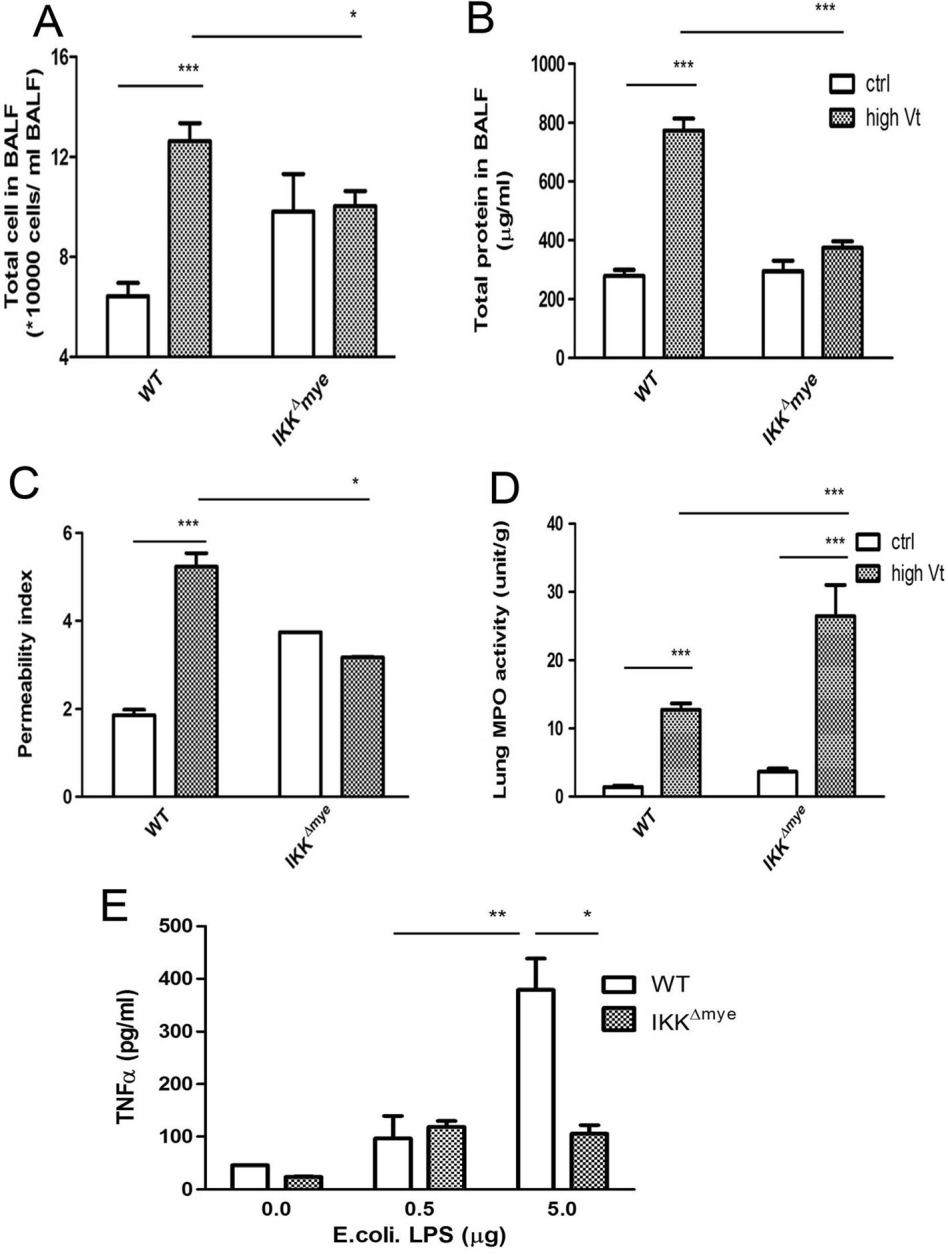
**Decreased ventilator-induced lung damage in IKKβ**^**△mye **^**mice.** High tidal volume ventilation induced total number of cells in BALF **(A)**, protein concentration in BALF **(B)**, and pulmonary microvascular permeability **(C)** in WT mice but not in IKKβ^△mye^ mice. However, there was more neutrophil sequestration in the IKKβ^△mye^ mice **(D)**. *P<0.05, **P<0.01, ***P<0.001. **(E)**. E.coli. LPS stimulation-induced activation of alveolar macrophages was decreased in IKKβ^△mye^ mice when compared with WT mice.

Both clinical and experimental studies have revealed that VILI pathogenesis involves triggering the inadvertent activation of inflammation [24]. NF-κB is an important nucleoprotein for the regulation of both the innate and adaptive immune responses. To evaluate the mechanism of NF-κB activation in VILI pathogenesis, mice with IкB kinase deletion in the myeloid cells (IKKβ^△mye^) were used. After high-stretch ventilation; the total number of cells in BALF, total protein in BALF, and pulmonary vascular permeability had significant 20%, 50%, and 40% decreases, respectively, (Figure 3A, 3B, 3C) in IKKβ^△mye^ mice compared to WT mice. However, more neutrophils were sequestered in the lung of IKKβ^△mye^ mice with ventilator treatment than the WT mice (Figure 3D). This indicates that NF-κB activation in the myeloid cells decreases ventilator-induced neutrophil infiltration in the lung.

### Decreased alveolar macrophage activity in IKKβ^△mye^ mice

The alveolar macrophages of IKKβ^△mye^ mice showed a 70% decrease in TNF-α production compared to WT mice when stimulated with high dose LPS (Figure 3E). NF-κB activation in the myeloid cells is thus important for the activation of alveolar macrophages.

### Ventilator-induced IL-6 production was significantly decreased in IKKβ^△mye^ mice

**Figure 4:**
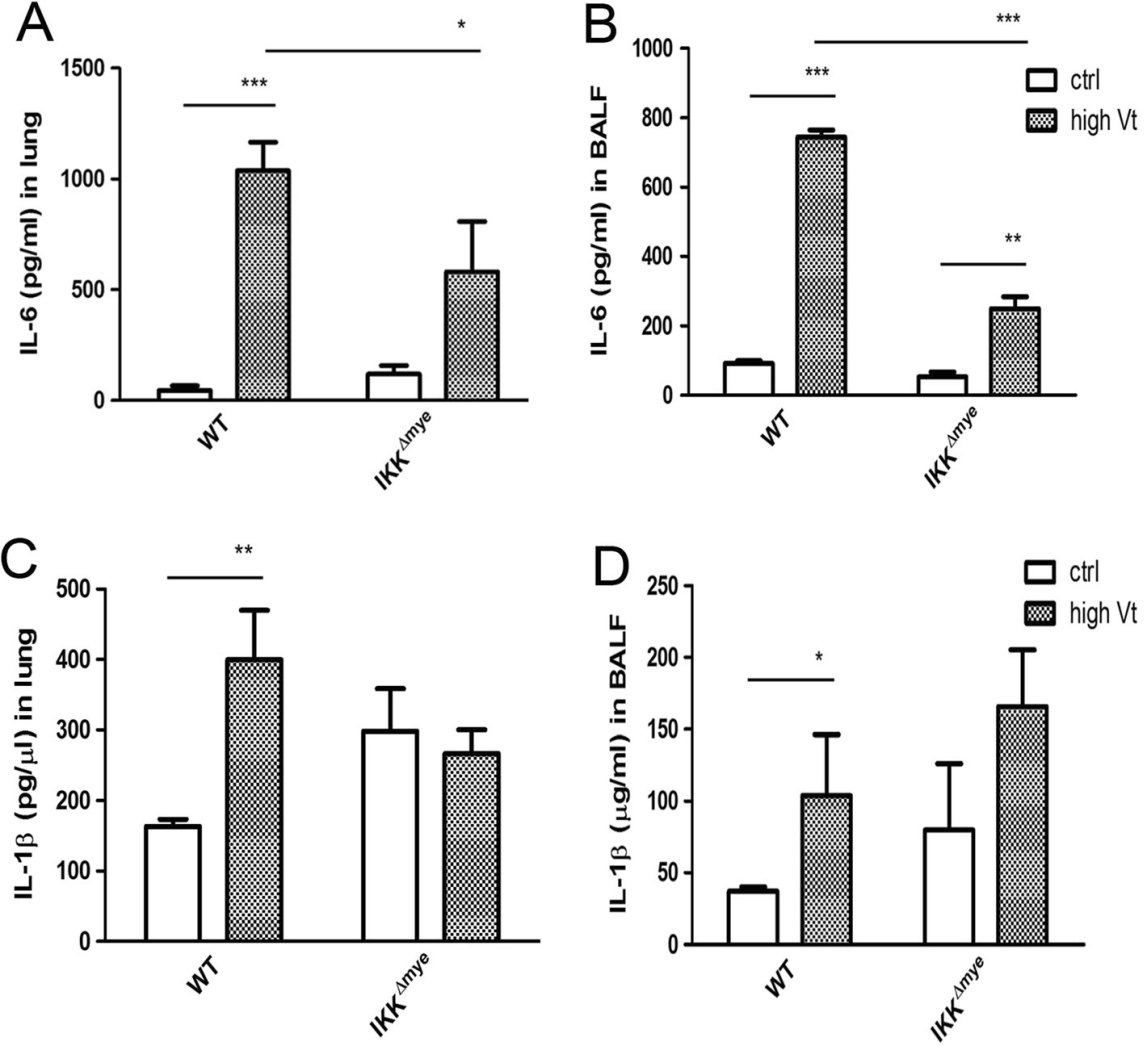
**Levels of proinflammatory cytokines in the lung and BALF in IKKβ**^**△mye **^**mice.** High tidal volume ventilation-induced IL-6 levels in the lung **(A)** and BALF **(B)** were decreased in IKKβ^△mye^ mice when compared with WT mice. IL-1β levels were assayed in the lung **(C)** and BALF **(D)** of IKKβ^△mye^ mice and WT mice. *P<0.05, **P<0.01.

The levels of proinflammatory cytokines, IL-6 and IL-1β, in the lung and BALF of IKKβ^△mye^ mice were also found to contribute to NF-κB activation in ventilator-induced IL-6 and IL-1β production. Significant 50% and 65% decreases in ventilation-induced IL-6 levels in the lung and BALF, respectively, were observed in IKKβ^△mye^ mice compared to WT mice (Figure 4A, 4B). High tidal volume ventilator treatment did not alter IL-1β levels in IKKβ^△mye^ mice (Figure 4C). This indicates that NF-κB activation in the myeloid cells plays an important role in ventilator-induced IL-6 production in the lung and BALF.

### IL-6-blocking antibody treatment decreased ventilator-induced IL-1β production

**Figure 5:**
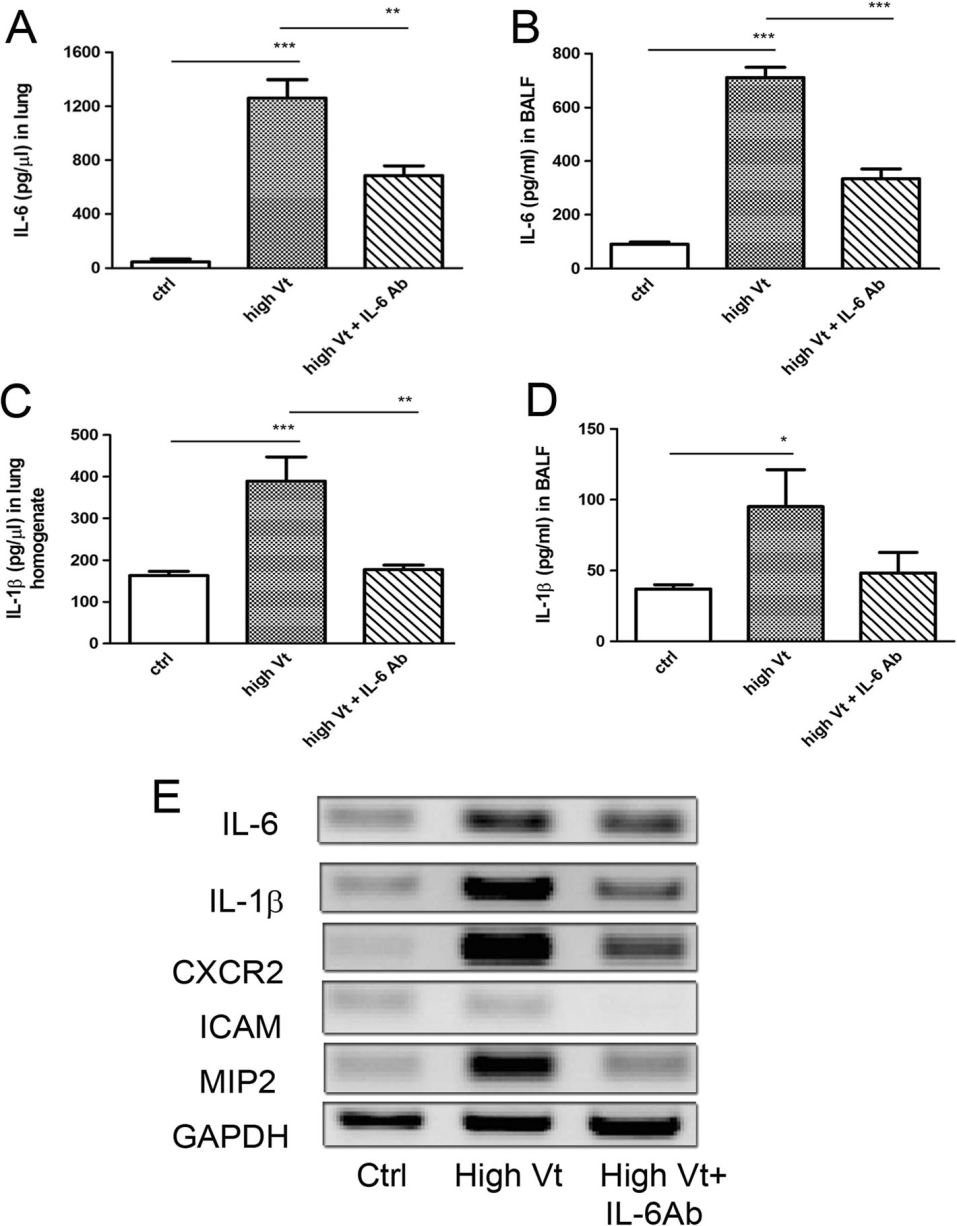
**Mechanical ventilation induced a significant increase of cytokines/chemokines in WT mice but not in IL-6 antibody-treated WT mice.** IL-6 antibody pretreatment decreased high tidal volume ventilation-induced IL-6 levels in the lung **(A)** and BALF **(B)** of WT mice. IL-6 antibody pretreatment decreased high tidal volume ventilation-induced IL-1β levels in the lung **(C)** and BALF **(D)** of WT mice. *P<*0*.*05*, **P<*0*.*01*, ***P<*0*.*001*. **(E)** IL-6 antibody treatment decreased high stretch ventilation-induced mRNA expression of proinflammatory cytokines (IL-6, IL-1β) and chemokines (CXCR2, ICAM, MIP-2) in the lung.

Levels of IL-6 in the lung and BALF have been shown to be significantly increased after ventilator treatment. Therefore, a specific IL-6-blocking antibody was used to study the role of IL-6 in VILI. Besides the significant decrease of IL-6 levels in the lung and BALF (Figure 5A, 5B), injection of IL-6-blocking antibody before high-stretch ventilation procedure markedly decreased the IL-1β production in the lung and BALF (Figure 5C, 5D). Moreover, IL-6-blocking antibody treatment significantly decreased ventilator-induced IL-1β, CXCR2, and MIP2 mRNA expression in the lung (Figure 5E). This indicates that specific IL-6-blocking antibody treatment prevents ventilator-induced IL-1β, CXCR2, and MIP2 expression in the lung.

### IL-6-blocking antibody treatment decreased ventilator-induced lung injury

**Figure 6:**
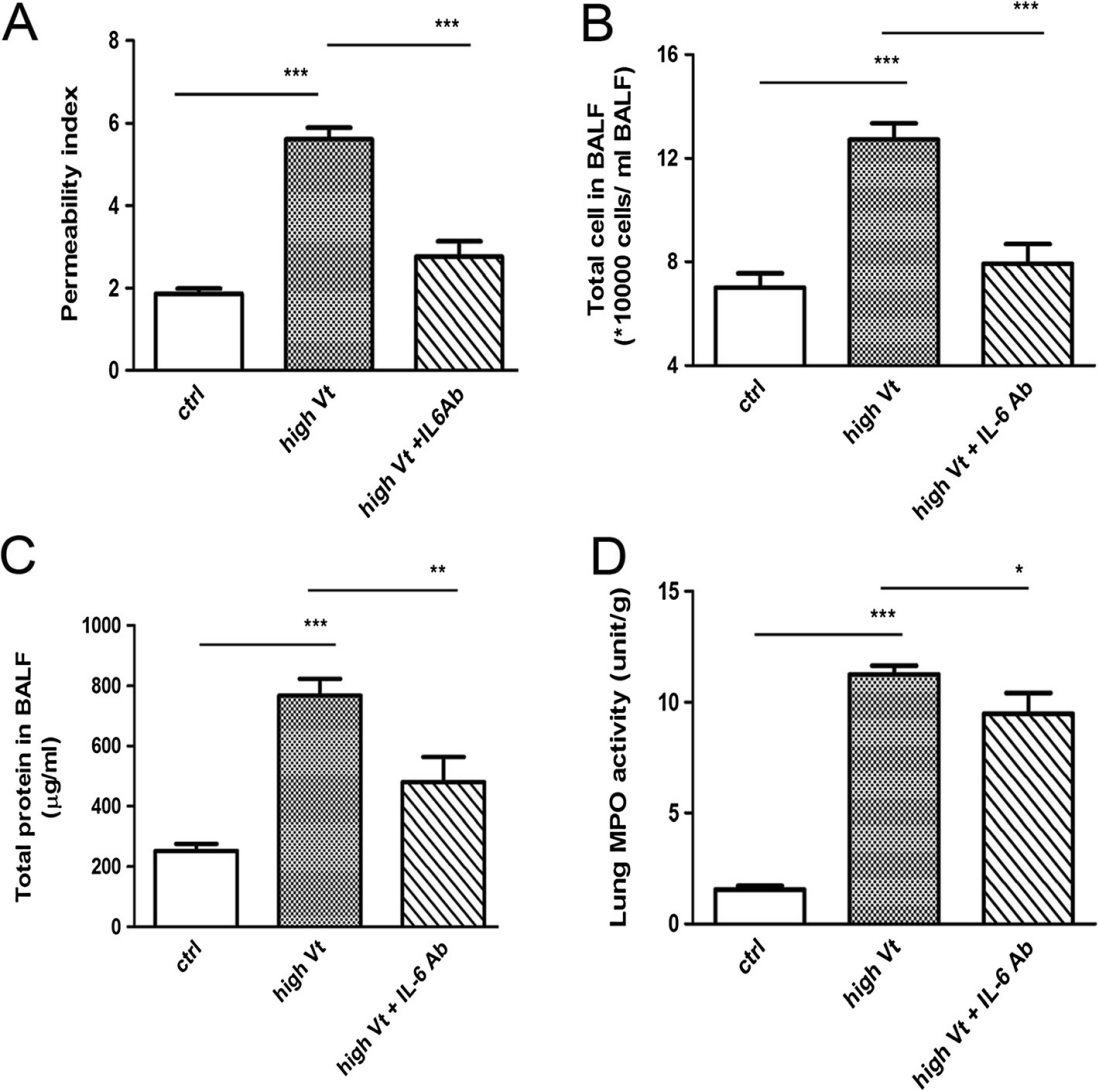
**Effect of IL-6-blocking antibody pretreatment on high tidal volume ventilation-induced lung damage in WT mice.** The pulmonary microvascular permeability **(A)**, total number of cells in BALF **(B)**, total protein in BALF **(C)** and neutrophil sequestration **(D)** in BALF were determined in WT mice with IL-6-blocking antibody treatment. *P<0.05, **P<0.01, ***P<0.001.

Intraperitoneal injection of IL-6-blocking antibodies in WT mice reversed the effects of ventilation on pulmonary vascular permeability and total number of cells in BALF (Figure 6A, 6B). Also, IL-6-blocking antibody treatment caused significant 15% and 40% decreases in pulmonary neutrophil sequestration and total protein concentration in BALF (Figure 6C, 6D), respectively, when compared to the high tidal volume group. This indicates that IL-6-blocking antibody treatment decreases high tidal volume ventilator-induced lung injury.

### IL-6^-/-^ to WT rather than WT to WT chimeric mice showed a significant decrease in ventilator-induced lung injury

**Figure 7:**
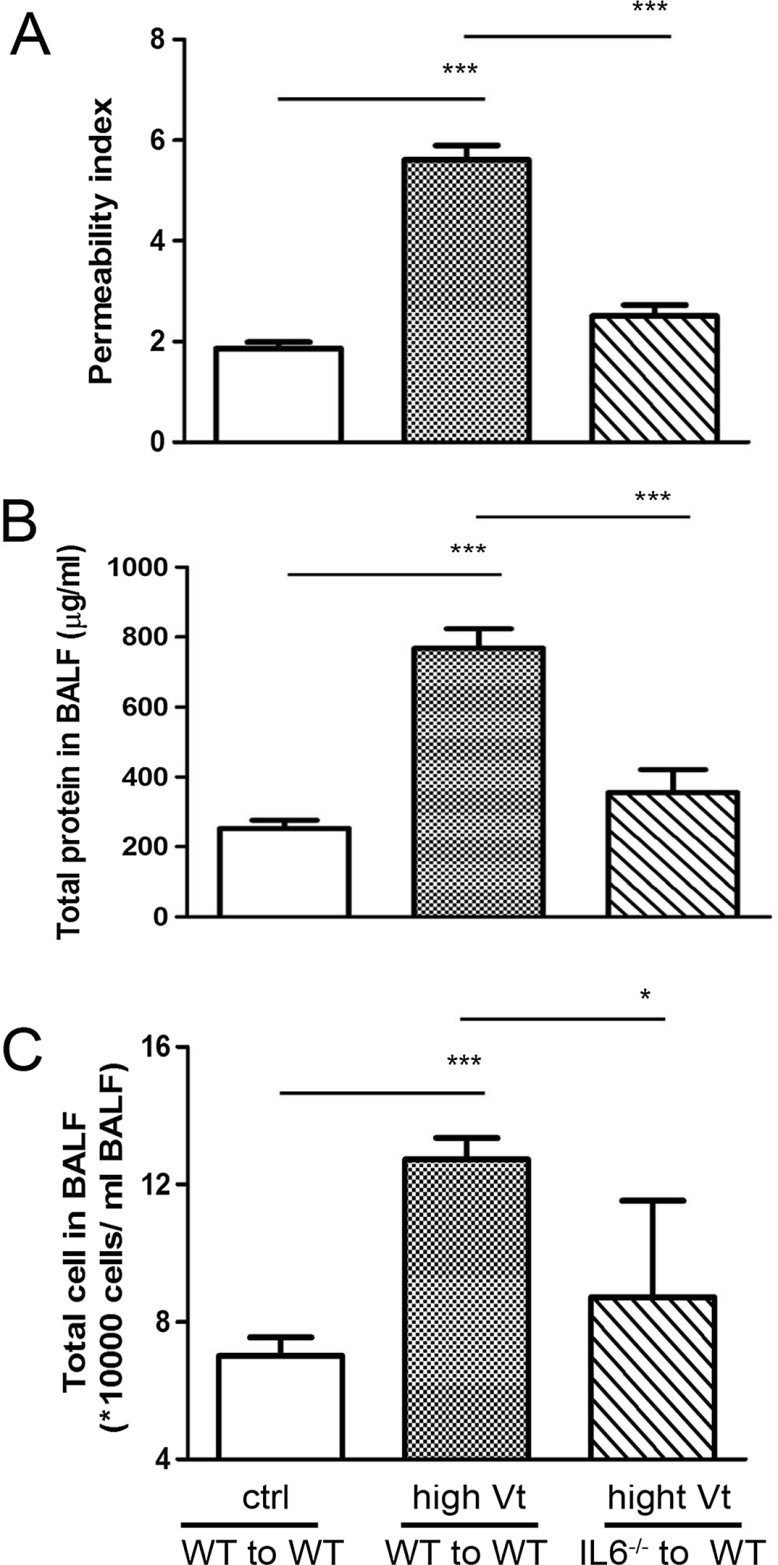
**Decreased ventilator-induced lung damage in IL6**^**-/- **^**to WT chimeric mice but not in WT to WT mice.** High tidal volume ventilation induced pulmonary microvascular permeability **(A)**, protein concentration of BALF **(B)**, and total number of cells in BALF **(C)** in WT to WT mice but not in IL6^-/-^ to WT chimeric mice. *P<0.05, **P<0.01, ***P<0.001.

To determine whether IL-6 on the myeloid cells plays a major role in the ventilator-induced lung injury, we harvested bone marrow cells from WT and IL-6^-/-^ mice and injected them into lethally irradiated WT mice respectively to generate WT to WT mice and IL-6^-/-^ to WT mice. The extent of ventilator-induced pulmonary vascular permeability, protein concentration as well as total cell count in BALF were all significantly attenuated in IL6^-/-^ to WT chimeric mice when compared to WT to WT mice (Figure 7A, 7B, 7C). This indicates that IL-6 on the myeloid cells plays an important role in high tidal volume ventilator-induced lung injury.

## Discussion

The pathogenesis and molecular mechanisms of ventilator-associated lung injury remain elusive. Clinical studies have shown that additional end-expiratory pressures and lower tidal volume can be protective and produce a better prognosis [25]. However, in particular situations, this protective ventilation strategy is insufficient to improve pulmonary gas exchange in ill patients. The stretch of ventilation may result in alveolar overdistention, and subsequently induces barotrauma, volutrauma, and biotrauma of lung.

In this study, an animal model of VILI was established and the lung damage was induced in WT mice through ventilation. First, the effects of low and high tidal volume were examined in WT mice. There were significant increases in pulmonary microvascular permeability and neutrophil infiltration in mice after ventilation, indicating that disruption of alveolar barriers can result in leakage of inflammatory substances and leukocytes. The increased vascular permeability and neutrophil infiltration led to pulmonary edema and a decline in lung function. With VILI progression, the released cytokines/chemokines promote neutrophil adhesion and sequestration to the lung [26]. These inflammatory responses could cause additional lung damage under the stimulation of ventilation. Collection of bronchoalveolar lavage fluid (BALF) is a common medical procedure to assay drifting leukocytes and the released proteins in the alveolar space, that can be used to diagnose lung diseases. The BALF was assayed for the total cell counts and protein concentration to represent the progressive lung damage with increased tidal volume. The levels of proinflammatory cytokines IL-6 and IL-1β in BALF of WT mice with ventilator treatment were higher than in the control group. Also, both mRNA and protein levels of the proinflammatory cytokines (IL-6 and IL-1β) and chemokines (CXCR2 and MIP-2) were increased after ventilation. In addition, NF-κB, the network hub of immunity and inflammation, was also activated after ventilator treatment and activated NF-κB can trigger a series of inflammatory cascades. The extent of VILI was also observed in the histological morphology showing the swelling of parenchyma and alveoli as well as altered cells staining in ventilated WT mice.

To investigate the involvement of NF-κB activation of myeloid cells in VILI, the IKKβ^△mye^ mice were used. The pulmonary microvascular permeability, total cell number and protein concentration in BALF, and alveolar macrophage activity were significantly decreased in IKKβ^△mye^ mice after high-stretch ventilation compared to WT mice. However, there was more neutrophil infiltration in the lungs of IKKβ^△mye^ mice. Recently, it had been demonstrated that IKKβ^△mye^ mice would develop neutrophilia and have higher neutrophil counts in their blood [27]. Our data further suggest that IKKβ in myeloid cells plays an important role in inducing the activity of alveolar macrophages and decreasing the ventilator-induced neutrophil infiltration in the lung. In addition, despite unchanged IL-1β expression, IKKβ^△mye^ mice with ventilator treatment produced markedly decreased levels of IL-6 in the lung and BALF when compared with WT mice. Moreover, IL6^-/-^ to WT but not WT to WT chimeric mice demonstrated a significant decrease in ventilator-induced lung damage. Altogether, these suggest that VILI depends on NF-κB activation in the myeloid cells and subsequent IL-6 production. Inhibition of NF-κB activation reduces IL-6 production and blocks the inadvertent inflammation cascade that contributes to ventilator-induced lung injury.

Although IL-6 was substantially increased among the proinflammatory substances examined in the ventilator model, the critical role of this pleiotropic cytokine in VILI is still controversial. A previous study found that IL-6 provides a protective effect in hyperoxic acute lung injury and VILI by reducing mortality, protein leakage, and endothelial and epithelial membrane injury through decreasing cell death and DNA fragmentation [15]. In contrast, it was reported that IL-6 beneficially limited the disruption of alveolar barrier and regulated neutrophils adhesion and migration [8]. However, elevated IL-6 levels have been observed in most experimental VILI models and IL-6 can be a biological maker of VALI [6,16]. In this study, the steady increase of IL-6 levels in the lung and BALF were observed after ventilation as demonstrated by mRNA or protein detection. To investigate the role of IL-6 in this VILI model, a specific IL-6-blocking antibody (0.25 mg/kg) was intraperitoneally injected to WT mice just before high-stretch ventilation, which had significant therapeutic effects in the arthritis [28]. After 6 hr of ventilation procedure, mice pretreated with IL-6-blocking antibodies showed a decrease in proinflammatory cytokines and adhesion molecules when compared with high tidal volume group. Besides, blocking IL-6 production in VILI had positive effects as demonstrated by decreased lung damage. This suggests that IL-6 production in the lung plays an important role in ventilator-induced IL-1β, CXCR2, as well as MIP2 production and subsequent lung injury. Furthermore, ventilator-induced pulmonary vascular permeability, protein concentration as well as total cell count in BALF were all significantly decreased in IL6^-/-^ to WT but not in WT to WT chimeric mice. This indicates that IL-6 on the myeloid cells plays an important role in high tidal volume ventilator-induced lung injury. Moreover, this further corroborates the finding that ventilator-induced lung injury through the NF-κB-IL-6 signaling pathways in myeloid cells. Using IL-6 or NF-κB inhibitors could be a useful strategy for decreasing mechanical ventilation-induced lung injury in respiratory failure patients.

## Conclusions

Mechanical ventilation induces significant increases in neutrophil accumulation, proinflammatory cytokines in the lung, total cells as well as protein in BALF, and pulmonary permeability in WT mice. However, the indicators of lung injury were decreased in WT mice receiving IL-6-blocking antibodies as well as in IL6^-/-^ to WT chimeric mice. Also, decreased IL-6 levels and VILI in IKKβ^△mye^ mice suggests that NF-κB activation-induced IL-6 expression potentially contributes to VILI pathogenesis. Therefore, NF-κB inhibitors may be useful in decreasing high tidal volume ventilation-induced IL-6 production and lung injury.

## Competing interests

The authors declare that they have no competing interests.

## Authors’ contributions

CMH and LWC designed the research. YAK and MCY performed the research. HTH analyzed the data. YAK, CMH and LWC wrote the article. All authors read and approved the final manuscript.
